# Revisiting gall anatomy through a functional-trait approach across environmental gradients

**DOI:** 10.1093/aobpla/plag038

**Published:** 2026-08-27

**Authors:** Filipe Gomes-Silva, Renê Gonçalves Silva Carneiro

**Affiliations:** Programa de Pós-Graduação em Ecologia e Evolução, Instituto de Ciências Biológicas, Universidade Federal de Goiás, Avenida Esperança, s/n, bloco ICB5, Câmpus Samambaia, Goiânia, Goiás 74690-900, Brasil; Departamento de Botânica, Instituto de Ciências Biológicas, Universidade Federal de Goiás, Avenida Esperança s/n, bloco ICB1, Câmpus Samambaia, Goiânia, Goiás 74690-900, Brasil

**Keywords:** tissue investment index, morphofunctional profiles, neotropical galls, plant–gall interactions

## Abstract

Different gall morphotypes reflect the reactivity of host plants and the specificity of their interactions with gall-inducing organisms. Gall structure is functionally divided into internal and external tissue compartments, which play nutritional and protective roles, respectively. We propose that gall structure is functionally responsive to environmental gradients, with a trade-off between investment in external and internal compartments that varies consistently with environmental cues. Furthermore, we also expect to identify patterns in the expression of anatomical traits across different environments. We compiled literature data on the anatomy and structure of Neotropical galls, standardized gall traits, and quantified tissue investment in each compartment using a Tissue Investment Index (TII). Then, we applied multivariate methods and hierarchical clustering to classify morphotypes into distinct morphofunctional profiles, followed by redundancy analysis and generalized linear models to relate these profiles to climatic variables. Anatomical traits were distributed across broad morphofunctional groups oriented towards protection or nutrition, with humidity and solar radiation emerging as the environmental variables most consistently associated with morphoanatomical variation, together explaining approximately 16% of total trait variation. Humidity was primarily associated with the degree of cellular hypertrophy and hyperplasia and with investment in the internal compartment, which was relatively higher under greater water availability. Solar radiation was associated with parenchyma homogenization and epidermal thickness, traits linked to water retention and metabolic capacity under higher irradiance. These patterns were consistent across diverse plant–gall systems, suggesting that gall anatomical traits respond to environmental gradients and may serve as informative indicators of habitat conditions.

## Teaser

Some insects manipulate plant tissues to build galls: structures that protect and feed the developing inducer. By comparing gall anatomy across many Neotropical species, we found that galls balance protective and nutritive tissue investment depending on local humidity and solar radiation. This trade-off emerges across insect-plant systems, suggesting that gall structure aligns with environmental conditions and could serve as a simple biological indicator of habitat quality.

## Introduction

Plants have the extraordinary capacity to perceive environmental cues and respond variably to abiotic pressures such as light, temperature, and humidity, but also to biotic pressures such as herbivory and parasitism (Sultan 2000, Agrawal 2001, Nicotra et al. 2010). However, multiple organism lineages independently evolved the capacity to manipulate plant anatomy and physiology, giving rise to galls: nutritive and protective structures composed entirely of plant tissues (Stone and Schönrogge 2003, Oliveira et al. 2016, Isaias et al. 2024). The close evolutionary relationships between gall-inducing insects and their host plants, characterized by different degrees of specialization that range from monophagy to more flexible forms of host use, result in unique gall morphologies that can be associated with each taxon, since their development occurs under the influence of the inducing organism (Espírito-Santo and Fernandes 2007, Joy and Crespi 2007, Hardy et al. 2020). Additionally, pressures from parasitoids and inquilines shape gall traits, i.e. morphology, color, and internal architecture, favoring adaptations that enhance inducer survival (Price et al. 1987, Bailey et al. 2009, Gonçalves et al. 2025). Beyond cues from inducers and their associated guilds, variation in host-organ traits may also contribute to gall morphoanatomical development (Cardoso et al. 2023). Thus, gall morphological diversity likely emerges from complex interactions among host developmental plasticity, inducer manipulation strategies, and environmental conditions (Ferreira et al. 2019).

Gall diversity is also associated with varying degrees of structural complexity. In structurally complex galls, two distinct morphofunctional compartments are typically recognized: an inner compartment, involved in inducer nutrition and composed of nutritive or nutritive-like tissues (NT/NLT), and an outer compartment, responsible for protection and storage, formed by parenchymatous and/or sclerenchymatous tissues and externally covered by epidermis or periderm (Bragança et al. 2017, Ferreira et al. 2017, 2019, Isaias et al. 2024). These compartments are often separated by a sclerenchymatous sheath and/or by vascularized median cortical layers (Ferreira et al. 2019). Because nutritive tissues are rarely vascularized, they depend on cell-to-cell transport of water, nutrients, and organic compounds, underscoring the importance of vascular access in sustaining metabolic gradients, driving cell redifferentiation, and maintaining the gall (Carneiro and Isaias 2015, Ferreira et al. 2019, Bragança et al. 2020). Thus, gall compartmentalization reflects a functionally integrated tissue organization shaped both by the inducer’s demands, which primarily affect the inner compartment, and by environmental conditions, which correlate with traits of the outer compartment (Costa et al. 2022, Fernandes et al. 2026).

The morphological and anatomical diversity of galls reported in the literature underscores the influence of abiotic factors, particularly in xeric environments characterized by water scarcity, high temperatures, and nutrient-poor soils, which produce galls with increased tissue thickness and accumulation of defensive compounds (Fernandes et al. 2026). This evidence supports both the environmental stress hypothesis, which predicts an increase in gall richness and abundance due to aridity-driven reductions in natural enemy pressures (Fernandes and Price 1988, Cuevas-Reyes et al. 2004), and the microenvironmental hypothesis, which frames galls as protective hygrothermal microhabitats (Price et al. 1987). Variations in soil chemistry (e.g. iron), gradients of water stress, and variations in light incidence have been shown to directly modulate gall traits such as cell-wall thickness, outer-compartment development, and pigment accumulation, indicating that galls are capable of being, to a certain extent, phenotypically plastic (Palacio-López et al. 2015, Bomfim et al. 2019, Arriola et al. 2024, Fernandes et al. 2026). In fact, galls formed on highly plastic organs like leaves tend to exhibit greater structural variability than those on less plastic organs such as stems (Formiga et al. 2015), evidencing the importance of plant phenotypic plasticity for gall development.

Anatomical variations of galls reinforce their value as model systems for studying parasite–host interactions across ecological gradients, even though the mechanisms regulating gall morphological diversity and spatial distribution remain only partially understood (Isaias et al. 2012). Based on the notion that morphofunctional compartments of galls display varying degrees of anatomical complexity, being responsive to external stimuli, we hypothesized that the expression of gall anatomical traits—particularly those of the external compartment—can also be modulated by abiotic environmental factors. Specifically, we predict that in more stressful environments, characterized by lower precipitation and higher levels of light irradiance and temperatures, galls will develop more pronounced external tissue compartments associated with protection and storage, reflecting greater relative allocation of tissue thickness to this compartment, rather than necessarily a lower metabolic cost of building it. Conversely, in less stressful environments, investment is expected to shift towards more developed internal compartments, involved in nutrition. We interpret this anatomical adjustment as a functional trade-off between defence and nutrition, analogous to trade-offs observed in other plant structures subjected to gradients of environmental stress (Franks and Farquhar 2007, Nicotra et al. 2010, Gratani 2014). This has been previously tested by Fernandes et al. (2026) for *Caryocar brasiliense* Cambess. and its associated galls subjected to drying environments, and here, we propose to expand the testing of this hypothesis, using anatomical data compiled from the specialized literature and analysing it on a broader scale to understand how the environment may shape gall traits across different plant–gall systems.

## Methods

### Data compilation

A systematic literature search was performed on 24 November 2023, using the Web of Science and Scopus databases with the keywords ‘gall’ AND ‘anatomy’ AND ‘structure’. Only studies that included histological images of leaf galls were selected, provided they allowed for the identification of anatomical traits. To ensure consistency, only galls in the mature developmental stage were considered. This strategy was adopted to prioritize specificity in the retrieval of studies explicitly addressing gall anatomical structure, ensuring that included records contained sufficient histological detail for standardized trait extraction and comparative analysis. Qualitative anatomical characters were recorded when clearly identifiable and standardized in terminology and concept according to Ferreira et al. (2019) (Table 1). The following traits were evaluated to build a presence/absence matrix: (i) indumentum; (ii) thick cuticle; (iii) thick epidermis/periderm; (iv) sclerenchyma; (v) high degree of cell hypertrophy; (vi) high degree of hyperplasia; (viii) high degree of parenchyma homogenization (see protocol for obtaining data in Table 2). For quantitative analysis, tissue compartments were defined based on the position of vascular bundles, which served as anatomical landmarks separating the external compartment (EC) formed by a common storage tissue (CST) from the internal compartment formed by nutritive or nutritive-like tissues.

**Table 1:** Matrix of presence/absence of anatomical traits for each gall morphotype per referenced study.

References	Host plants	Gall inducers	Morphotype	A	B	C	D	E	F	G	TII	LAT	LONG	ALT	Location/Country
Scareli-Santos et al. (1999)	*Anadenanthera peregrina* (L.) Speg.	*Schizomyia* sp.	Conical	+	+	−	+	−	−	−	5.146	−47.866	−21.966	878	São Carlos/BR
Scareli-Santos et al. (1999)	*Anadenanthera peregrina* (L.) Speg.	*Schizomyia* sp.	Globoid	+	+	−	+	−	−	−	8.085	−47.866	−21.966	878	São Carlos/BR
Souza et al. (2000)	*Ficus microcarpa* L.f.	*Gynaikothrips ficorum*	Leaf fold	+	+	−	−	+	+	−	0.748	−47.898	−22.004	822	São Paulo/BR
Scareli-Santos and Varanda (2003a)	*Duguetia furfuracea* (A.St.-Hil.) Saff.	Cecidomyiidae	Globoid	+	+	−	+	−	−	−	7.775	−47.583	−21.583	689	Santa Rita do Passa Quatro/BR
Scareli-Santos and Varanda (2003a)	*Duguetia furfuracea* (A.St.-Hil.) Saff.	Cecidomyiidae	Polypoid	−	+	−	−	−	−	−	6.970	−47.583	−21.583	689	Santa Rita do Passa Quatro/BR
Scareli-Santos and Varanda (2003a)	*Duguetia furfuracea* (A.St.-Hil.) Saff.	Cecidomyiidae	Lenticular	+	+	−	+	−	−	−	1.490	−47.583	−21.583	689	Santa Rita do Passa Quatro/BR
Scareli-Santos and Varanda (2003b)	*Tabebuia ochracea* (Cham.) Standl.	Cecidomyiidae	Globoid	+	+	−	+	+	+	−	3.041	−47.583	−21.583	689	Santa Rita do Passa Quatro/BR
Oliveira et al. (2006)	*Lonchocarpus muehlbergianus* Hassl.	*Euphalerus ostreoides*	Bivalve shaped	+	−	−	+	+	+	−	3.322	−43.972	−19.874	845	Belo Horizonte/BR
Oliveira and Isaias (2010)	*Copaifera langsdorffii* Desf.	—	Lenticular	−	+	−	−	+	+	+	2.000	−43.983	−20.093	1486	Brumadinho
Agudelo et al. (2013)	*Schinus longifolius* (Lindl.) Speg.	*Calophya mammifex*	Bivalve shaped	+	−	−	+	−	−	−	2.329	−58.352	−34.607	5	Zárate/ARG
Carneiro et al. (2014)	*Psidium myrtoides* O.Berg	*Nothotrioza myrtoidis*	Globoid	−	−	+	+	+	+	−	3.038	−43.407	−20.074	754	Catas Altas/BR
Formiga et al. (2015)	*Baccharis reticularia* DC.	Psyllidae	Leaf roll	+	−	+	−	+	+	+	0.758	−43.516	−20.116	1497	Campo de Fora/BR
Formiga et al. (2015)	*Baccharis reticularia* DC.	Psyllidae	Kidney shaped	−	−	+	−	+	+	−	1.836	−43.516	−20.116	1497	Campo de Fora/BR
Formiga et al. (2015)	*Baccharis reticularia* DC.	Psyllidae	Fusiform	−	−	−	+	+	+	+	0.544	−43.516	−20.116	1497	Campo de Fora/BR
Formiga et al. (2015)	*Baccharis reticularia* DC.	Psyllidae	Globoid	−	+	+	+	+	+	+	0.422	−43.516	−20.116	1497	Campo de Fora/BR
Carneiro et al. (2015)	*Psidium cattleianum* Sabine	*Nothotrioza cattleiani*	Globoid	−	−	−	−	+	+	−	4.093	−48.916	−25.445	787	Piraquara/BR
Jorge et al. (2016)	*Myrcia retorta* Cambess.	*Holopothrips tabebuia*	Leaf roll	−	−	−	−	+	+	−	1.151	−48.55	−26.283	9	São Francisco do Sul/BR
Carneiro et al. (2017)	*Copaifera langsdorffii* Desf.	Cecidomyiidae	Horn shaped	+	−	−	−	+	+	−	1.244	−43.983	−20.093	1486	Brumadinho/BR
Corró Molas et al. (2017)	*Prosopis caldenia* Burkart	*Rhopalomyi*a sp.	Bivalve shaped	−	−	−	+	−	−	−	0.74	−64.301	−36.619	172	Santa Rosa/ARG
Aranda-Rickert et al. (2017)	*Prosopis chilensis* (Molina) Stuntz	*Eschatocerus acaciae*	Globoid	−	+	−	+	−	+	−	0.738	−66.943	−28.805	1393	Anillaco/ARG
Arriola et al. (2018)	*Smilax campestris* Griseb.	—	Globoid	+	−	+	+	+	−	+	1.193	−48.55	−26.283	9	Sao Francisco do Sul/BRA
Guedes et al. (2018)	*Schinus polygama* (Cav.) Cabrera	*Calophya mammifex*	Globoid	−	+	+	−	+	+	−	2.485	−72.278	−36.658	150	Bio-bio/CHL
Guedes et al. (2018)	*Schinus polygama* (Cav.) Cabrera	*Calophya rubra*	Conical	+	+	+	+	+	+	+	0.31	−72.278	−36.658	150	Bio-bio/CHL
Teixeira et al. (2018)	*Croton floribundus* Spreng.	*Clinodiplosis* sp.	Globoid	+	−	−	+	+	−	−	2.440	−44	−21	1062	Tiradentes/BRA
Jorge et al. (2018)	*Myrcia splendens* (Sw.) DC.	*Nexothrips* sp.	Leaf roll	−	−	−	−	+	−	−	1.656	−43.972	−19.874	845	Belo Horizonte/BRA
Agudelo et al. (2018)	*Baccharis spicata* (Lam.) Baill.	*Baccharopelma* sp.	Leaf fold	−	+	−	−	+	+	+	5.715	−58.352	−34.607	5	Buenos Aires/ARG
Oliveira et al. (2019)	*Pouteria ramiflora* (Mart.) Radlk.	*Ceropsylla pouteriae*	Lenticular	−	+	+	−	+	+	+	2.023	−48.306	−18.993	830	Uberlândia/BRA
Martini et al. (2019)	*Aspidosperma tomentosum* Mart.	*Pseudophacopteron longicaudatum*	Lenticular	−	−	−	−	+	+	−	3.431	−48.3	−19	855	Uberlândia/BRA
Silva et al. (2019)	*Matayba guianensis* Aubl.	*Bystracoccus mataybae*	Globoid	−	−	−	+	−	−	+	4.061	−48.3	−19	855	Uberlândia/BRA
Arriola and Isaias (2021)	*Miconia albicans* (Sw.) DC.	*Ditylenchus gallaeformans*	Globoid	+	−	+	−	−	−	+	0.916	−43.972	−19.874	845	Belo Horizonte/BRA
Arriola and Isaias (2021)	*Miconia ibaguensis* (Bonpl.) Triana	*Ditylenchus gallaeformans*	Globoid	+	−	+	−	−	−	−	1.792	−43.972	−19.874	845	Belo Horizonte/BRA
Arriola and Isaias (2021)	*Miconia corallina* Spring	*Ditylenchus gallaeformans*	Lenticular	−	+	+	−	−	−	+	2.685	−43.972	−19.874	1342	Ouro Preto/BRA
Arriola and Isaias (2021)	*Miconia lacunosa* (Cogn.) R.Goldenb.	*Ditylenchus gallaeformans*	Lenticular	+	−	−	−	−	−	−	1.414	−43.972	−19.874	1342	Ouro Preto/BRA
Farias et al. (2021)	*Cyathea phalerata* Mart.	Cecidomyiidae	Lenticular	−	−	−	−	+	+	−	3.248	−35.695	−8.498	680	Bonito/BRA
Guimarães et al. (2021)	*Clusia fluminensis* Planch. & Triana	Cecidomyiidae	Lenticular	−	+	+	+	+	−	−	1.264	−42.833	−22.883	23	Maricá/BRA
Costa et al. (2022)	*Mimosa gemmulata* Barneby	*Lopesia* sp.	Lenticular	+	−	−	+	−	−	+	6.572	−42.499	−14.076	937	Caetité/BRA
Costa et al. (2022)	*Mimosa gemmulata* Barneby	*Lopesia* sp.	Bivalve shaped	+	−	−	+	−	−	+	23.426	−42.499	−14.076	937	Caetité/BRA
Costa et al. (2022)	*Mimosa gemmulata* Barneby	*Lopesia* sp.	Bivalve shaped	+	−	−	+	+	−	+	12.522	−42.499	−14.076	937	Caetité/BRA
Costa et al. (2022)	*Mimosa gemmulata* Barneby	*Lopesia* sp.	Globoid	+	−	−	+	−	−	+	5.317	−42.499	−14.076	937	Caetité/BRA
Gonçalves et al. (2022)	*Matayba guianensis* Aubl.	*Lopesia mataybae*	Globoid	+	−	−	+	+	+	−	0.209	−48.4	−19.166	772	Uberlândia/BRA
Gonçalves et al. (2022)	*Matayba guianensis* Aubl.	*Lopesia mataybae*	Cylindrical	+	+	+	+	+	+	−	0.709	−48.4	−19.166	772	Uberlândia/BRA
Teixeira et al. (2022)	*Croton floribundus* Spreng.	Cecidomyiidae	Globoid	+	+	+	+	−	−	+	0.854	−44	−21	1062	Tiradentes/BRA
Teixeira et al. (2022)	*Croton floribundus* Spreng.	Cecidomyiidae	Globoid	+	−	−	+	−	−	+	1.167	−44	−21	1062	Tiradentes/BRA
Teixeira et al. (2022)	*Croton floribundus* Spreng.	Cecidomyiidae	Globoid	+	−	−	+	−	−	+	0.631	−44	−21	1062	Tiradentes/BRA
Teixeira et al. (2022)	*Croton floribundus* Spreng.	Cecidomyiidae	Globoid	+	−	−	−	−	−	+	1.506	−44	−21	1062	Tiradentes/BRA
Teixeira et al. (2022)	*Croton floribundus* Spreng.	Cecidomyiidae	Lenticular	+	−	+	+	−	−	+	2.073	−44	−21	1062	Tiradentes/BRA
Teixeira et al. (2022)	*Croton floribundus* Spreng.	Cecidomyiidae	Lenticular	+	−	+	−	−	−	−	6.364	−44	−21	1062	Tiradentes/BRA
Teixeira et al. (2022)	*Croton floribundus* Spreng.	Cecidomyiidae	Leaf roll	+	−	−	+	+	−	−	0.997	−44	−21	1062	Tiradentes/BRA
Teixeira et al. (2022)	*Croton floribundus* Spreng.	Cecidomyiidae	Fusiform	+	−	+	+	+	+	−	2.981	−44	−21	1062	Tiradentes/BRA
Nogueira et al. (2022)	*Mimosa tenuiflora* (Willd.) Poir.	*Lopesia mimosae*	Bivalve shaped	+	−	−	+	+	+	−	2.753	−42.499	−14.076	937	Caetité/BRA
Castro et al. (2023)	*Eugenia uniflora* L.	*Clinodiplosis profusa*	Fusiform	−	+	+	+	+	+	+	1.750	−43.221	−22.843	18	Rio de Janeiro/BRA
Freitas et al. (2023)	*Miconia ibaguensis* (Bonpl.) Triana	Eriophyidae	Lenticular	+	+	+	+	+	+	+	0.488	−43.972	−19.874	845	Belo Horizonte/BRA
Freitas et al. (2023)	*Miconia notabilis* Triana	Eriophyidae	Lenticular	−	−	−	+	+	+	+	0.997	5.068	−75.483	2144	Manizales/COL
Guedes et al. (2023)	*Schinus polygama* (Cav.) Cabrera	*Andescecidium parrai*	Globoid	+	−	−	+	+	+	−	11.265	−72.439	−36.79	81	Ruble/CHL
Silva et al. (2023)	*Annona dolabripetala* Raddi	*Pseudotectoccocus rolliniae*	Globoid	+	−	−	+	−	−	−	3.023	−43.97	−19.874	866	Belo Horizonte/BRA
Silva et al. (2023)	*Annona dolabripetala* Raddi	*Pseudotectoccocus rolliniae*	Globoid	+	−	−	+	−	−	−	0.718	−43.97	−19.874	866	Belo Horizonte/BRA
Silva et al. (2023)	*Pseudobombax grandiflorum* (Cav.) A.Robyns	*Eriogallococcus isaias*	Conical	+	−	−	+	−	−	−	1.280	−43.97	−19.874	866	Belo Horizonte/BRA
Silva et al. (2023)	*Pseudobombax grandiflorum* (Cav.) A.Robyns	*Eriogallococcus isaias*	Conical	+	−	−	+	−	−	−	0.925	−43.97	−19.874	866	Belo Horizonte/BRA
Bragança et al. (2023)	*Niphidium crassifolium* (L.) Lellinger	Cecidomyiidae	Globoid	−	−	−	−	+	+	−	1.245	−40.918	−20.173	1089	Santa Maria de Jetibá/BRA
Guedes et al. (2023)	*Tilia platyphyllos* Scop.	*Eriophyes tiliae*	Clavate	+	−	−	−	−	−	−	2.399	−73.035	−36.826	61	Concepción/CHL

(A) Indumentum, (B) thick cuticle; (C) thick epidermis/periderm; (D) sclerenchyma; (E) high degree of cell hypertrophy; (F) high degree of hyperplasia; (G) high degree of parenchyma homogenization; TII: Tissue Investment Index.

**Table 2:** Protocol for obtaining functional traits for the preparation of the traits matrix.

Trait	Code	Data type	Visual indicator	Textual indicator
Indumentum	Ind	Binary (presence/absence)	Identification in histological image	‘Trichome present’; ‘glandular hairs’
Thick cuticle	Cut	Binary (presence/absence)	Threshold application (≥1.5 µm)	‘thick cuticular layer’; ‘cutin thickening’; ‘reinforced cuticle’
Thick epidermis	Epi	Binary (presence/absence)	Threshold >1 cell layer for thick epidermis	‘multiseriate epidermis’; ‘polylayered epidermis’; ‘thick epidermis’
Sclerenchyma	Esc	Binary (presence/absence)	Presence or absence of a sclerenchymatous sheath	‘sclerenchymatous fibers’; ‘sclerenchymatous sheath’; ‘developed sclerenchymatous tissue’
High degree of cell hypertrophy	Htr	Binary (presence/absence)	Cell area increase ≥2×; giant/polynucleated cells	‘giant cells’; ‘expanded cells’; ‘polynucleated cells’; ‘pronounced hypertrophy’
High degree of hyperplasia	Hpl	Binary (presence/absence)	≥2 additional cell layers; intense cell multiplication	‘intense cell proliferation’; ‘significant increase in layers’; ‘accentuated cell division’
High degree of parenchyma homogenization	Hop	Binary (presence/absence)	≥ 2 layers of widely uniform parenchyma cells	‘uniform parenchyma texture’; ‘homogeneous parenchymatous structure’; ‘constant cell density’
Tissue investment index	TII	Quantitative	Ratio between external and internal compartment thickness	—

Thickness measurements were obtained from three different regions of each compartment: for the external compartment, measurements were taken from the outermost surface (the tips of trichomes or, when absent, the cuticle) to the inner edge of the vascular tissues; for the internal compartment, measurements extended from the inner edge of the vascular tissues to the innermost layers delimiting the larval chamber. The average thickness of each compartment was then calculated from the three measurements. Additionally, five measurements of individual cell areas were taken per compartment to estimate mean cell size. All measurements were taken from histological images analysed with digital image analysis tools (AxioVision 4.8; Carl Zeiss MicroImaging GmbH 2015) to ensure precision and reproducibility. The thickness measurements of the external and internal compartments of the galls were used to calculate a tissue investment index (TII) to assess the relative investment in each of the morphofunctional compartments. The index was obtained using the formula: TII = E/I, where E represents the thickness of the external compartment and I represents the thickness of the internal compartment. A TII value greater than 1 suggests a relatively greater structural allocation towards protective/storage tissues of the external compartment, while values less than 1 suggest a relatively superior investment in nutritive tissues of the internal compartment.

The Tissue Investment Index (TII) was designed as a comparative structural proxy to evaluate relative allocation patterns between gall compartments rather than as a direct measure of energetic expenditure. The index assumes that greater tissue development, expressed as compartment thickness, reflects increased developmental allocation and functional specialization associated with gall construction. However, because tissue types may differ in developmental complexity, metabolic costs, and ecological roles, TII should be interpreted as a relative indicator of structural allocation rather than an exact measure of tissue costs or functionality.

To minimize observer bias, all binary traits were independently scored by two authors, and discordant cases were resolved by consensus. We acknowledge that heterogeneity in histological procedures among source studies (e.g. magnification, staining protocols, section planes) represents an inherent limitation of literature-based comparative analyses and may introduce measurement error that cannot be fully controlled. This limitation is discussed further in the context of our results.

To compile the environmental dataset, all collection sites described in the selected studies were georeferenced (latitude, longitude, and collection period), and the relevant climatic variables were extracted for these coordinates. The variables included mean annual temperature, mean annual precipitation, mean relative humidity, and mean annual solar radiation (representing regional climatic conditions at the collection site rather than leaf-orcanopy-level light exposure), obtained from high-resolution climate databases. For collections conducted in Brazil, we used daily meteorological data from INMET (https://portal.inmet.gov.br/), which were aggregated into monthly and annual averages corresponding to the year of collection. For international locations, climatic data were obtained from the Climate Data Store of Copernicus (https://cds.climate.copernicus.eu/), using monthly averages for the 1991–2020 reference period. Because the datasets differed in temporal structure, all environmental variables were standardized using *z*-score transformation prior to multivariate analyses to ensure comparability among predictors and reduce scale differences. Although both sources provide equivalent climatic descriptors, we acknowledge that differences in temporal resolution and sampling framework may introduce uncertainty that cannot be fully controlled in literature-based comparative analyses.

### Data analysis

To investigate variation in gall anatomical traits and the Tissue Investment Index (TII), a trait matrix containing eight anatomical variables was constructed. Because the dataset included mixed variables, a Gower dissimilarity matrix was calculated (Gower 1971). We applied a log transformation to TII values to reduce skewness and mitigate the impact of outliers. A principal coordinates analysis (PCoA) was performed to ordinate the Gower dissimilarity matrix. The first two ordination axes were retained, and the proportion of variance explained by each axis was calculated from the eigenvalues.

To identify possible functional groupings among gall morphotypes, a hierarchical clustering analysis was applied. Hierarchical clustering was performed using Ward’s method based on Euclidean distances calculated from the scores of the first two PCoA axes. The morphotypes were classified into three clusters, and the composition of each group was described based on the mean values of the anatomical traits. Differences in TII among clusters were tested using the Kruskal–Wallis test.

Next, to evaluate the relationship between anatomical traits and environmental variables, we conducted a distance-based redundancy analysis (db-RDA) (Legendre and Anderson 1999), using the seven anatomical traits plus log-transformed TII as response and mean annual precipitation, temperature, relative humidity, and solar radiation as predictors. Before the analysis, the environmental variables were standardized to allow comparability among scales. Multicollinearity among them was tested using Pearson correlations.

Based on db-RDA results, which identified relative humidity and solar radiation as the main environmental gradients associated with anatomical variation (see Results), candidate generalized linear models (GLMs) and generalized linear mixed-effects models (GLMMs) with a Gamma error distribution and log link function were fitted to evaluate their effects on TII. Relative humidity and solar radiation were included as fixed effects, whereas gall–inducer family, host–plant family, and habitat type were initially evaluated as random intercepts in GLMMs to account for potential hierarchical dependencies in the data. Candidate models were compared using Akaike’s Information Criterion (AIC) (Akaike 1974), considering models with ΔAIC < 2 were considered to have substantial support (Burnham and Anderson 2002). To ensure comparability, model selection was performed using a common subset of observations with complete information for all candidate predictors and random-effect variables (see Supplementary Table S1). Because random effects contributed negligible additional explanatory power, the most parsimonious GLM was retained. The final GLM was subsequently refitted using all available observations with complete information for the retained predictors. Model fit was assessed through residual diagnostics, including dispersion tests, and 95% confidence intervals for model coefficients were estimated.

All analyses were conducted in R (R Core Team 2024) using the packages cluster (Maechler et al. 2023), vegan (Oksanen et al. 2020), glmmTMB (Brooks et al. 2017), DHARMa (Hartig 2021), and MASS (Venables and Ripley 2002).

## Results

The literature search returned 47 articles from Web of Science and 70 from Scopus, of which 36 met the inclusion criteria (Table 1), 19 from the first database and 17 from the second. Of these, 28 were from Brazil, with the majority concentrated in the Southeastern region, in addition to data found in Argentina, Chile and Colombia (Table 1). We observed that the diversity of gall morphotypes was directly proportional to the diversity of host plant species; however, the available data were mainly derived from localized studies, concentrated primarily in specific areas of the Neotropical region.

Based on our data, we identified plant species that host more than one gall morphotype. For example, *Mimosa gemmulata* (Fabaceae) exhibited a broader diversity of gall morphotypes such as lenticular, bivalve, and globoid; all induced by species of *Lopesia* (Cecidomyiidae) (Costa et al. 2022). In *Croton floribundus* (Euphorbiaceae), galls were induced by unidentified species of Cecidomyiidae and displayed remarkable morphological variation, including globoid, intralaminar and extralaminar lenticular, fusiform, and leaf-rolling galls (Teixeira et al. 2022). Particularly in the case of *Miconia* spp. (Melastomataceae), a single gall–inducer nematode, *Ditylenchus gallaeformans* (Anguinidae), is found associated with different hosts, inducing globoid galls in leaves (Arriola and Isaias 2021). Further details on the list of different plant–gall systems can be seen in Table 1.

**Figure 1:**
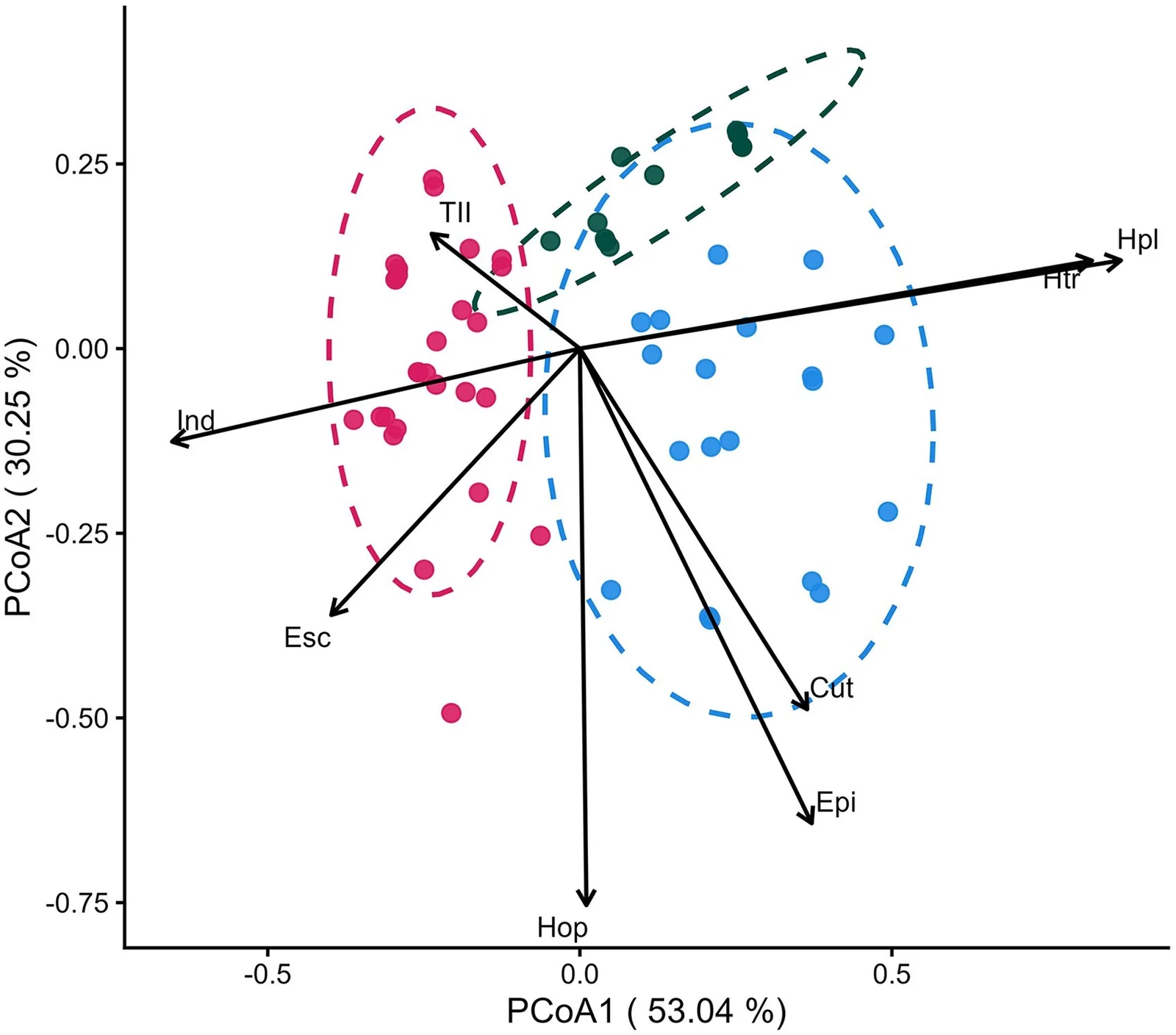
Principal coordinates analysis (PCoA) of seven binary anatomical traits and the log-transformed tissue investment Index (IT_log), based on Gower dissimilarity. The first two axes (PCoA1 = 53.04%, PCoA2 = 30.25%) are shown, with trait vectors greater than |0.2| displayed. PCoA1 contrasts galls with strong internal proliferation (hyperplasia, Hpl; hypertrophy, Htr) at positive scores against those with prominent external defences (indumentum, Indu; sclerenchyma, Esc) at negative scores. PCoA2 separates homogeneous parenchyma (Hop), thicker cuticle (Cut) and epidermis (Epi) at negative scores from tissue investment index (TII) at positive scores. Three clusters emerged: Cluster 1 (raspberry, PCoA1^−^/PCoA2^+^) comprises galls with high external defence and low internal proliferation; Cluster 2 (strong blue, PCoA1^+^/PCoA2^−^) exhibits both strong external reinforcement and internal proliferation; Cluster 3 (dark teal, PCoA1^+^/PCoA2^+^) is characterized by intense hyperplasia/hypertrophy with relatively homogeneous parenchyma. Abbreviations: Indu, indumentum; Cut, cuticle thickness; Epi, epidermal thickness; Esc, sclerenchyma; Htr, hypertrophy; Hpl, hyperplasia; Hop, parenchymal homogenization; TII, log-transformed Tissue Investment Index.

**Figure 2:**
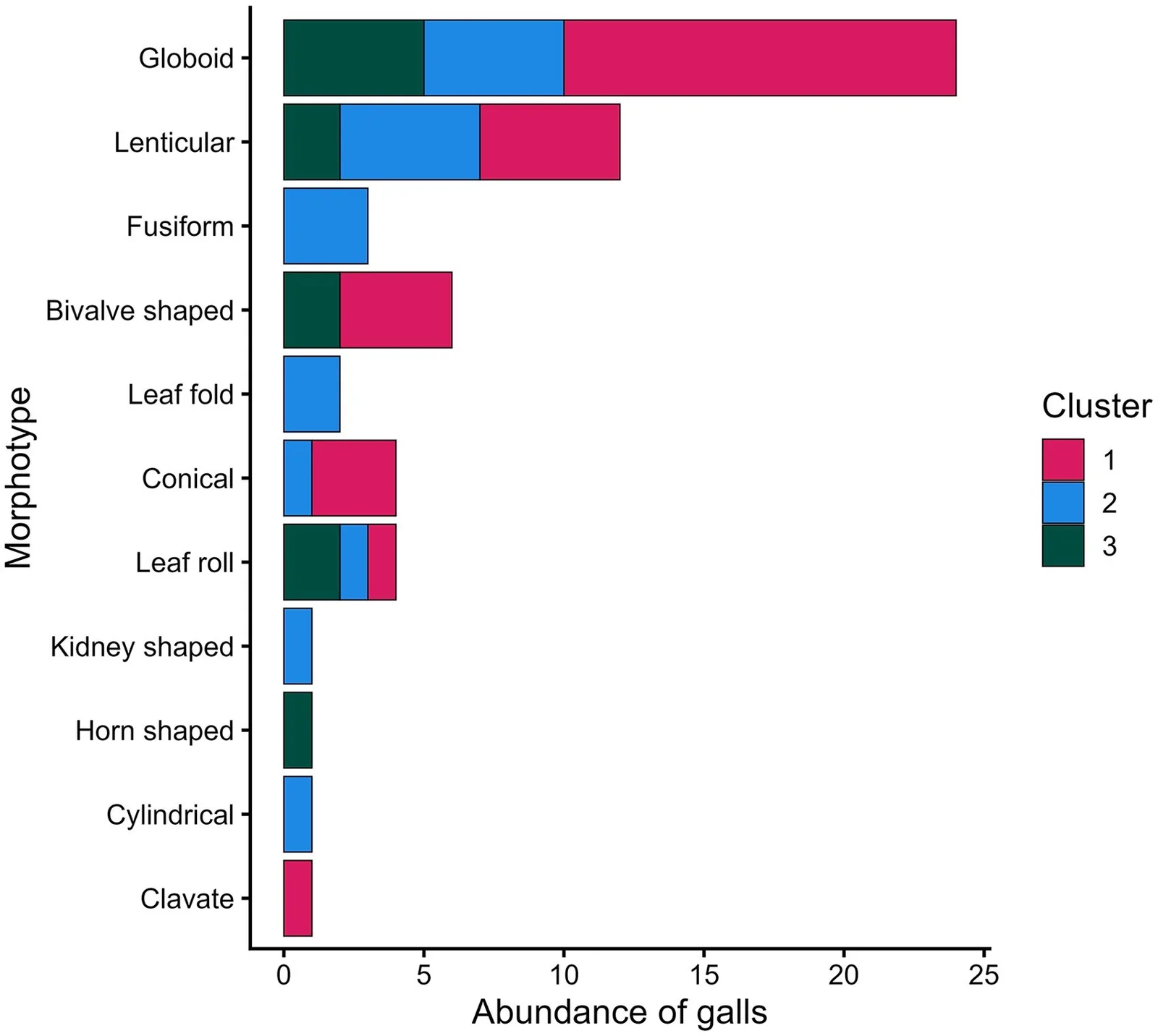
The abundance of gall morphotypes in each of the three PCoA-derived groups. Each bar represents a morphotype, and the segments of each bar correspond to clusters formed. The length of each segment indicates the number of galls of that morphotype within the group, illustrating the compositional differences between the groups.

In the ordination analysis, the degrees of cellular hyperplasia and hypertrophy were the main contributors to morphotype differentiation, with the first two principal coordinates explaining approximately 83% of the total variation, while the Tissue Investment Index showed a weaker association with the principal ordination axis (Fig. 1). These findings were corroborated by hierarchical clustering analysis, which identified three relatively cohesive groups differentiated based on their anatomical composition. The first group, composed primarily of globoid galls (Fig. 2), exhibited higher investment in the external compartment (mean TII = 1.28). These galls were characterized by the presence of trichomes and sclerenchyma, and, in contrast, the lowest degrees of cellular hyperplasia and hypertrophy compared to the other groups. The second group, with greater morphotype diversity and structural complexity, consisted of galls best defined by thicker cuticle and epidermis, and higher degrees of cellular hypertrophy and hyperplasia, combined with an overall lower investment in the external compartment (mean TII = 0.91). The third group also comprises a variety of gall morphotypes, and was distinguished by thinner cuticle and epidermis, less homogeneous parenchyma, along with an also higher external/internal tissue investment (mean TII = 1.29). The mean values of the Tissue Investment Index (TII) did not differ significantly among the three groups, such as evidenced by the Kruskal–Wallis test (*χ*^2^ = 4.18; df = 2; *P* = 0.124). Despite differences in anatomical composition among groups, TII showed a weaker association with the main ordination axis, suggesting that variation in tissue investment was not strongly structured by the cluster classification itself.

**Figure 3:**
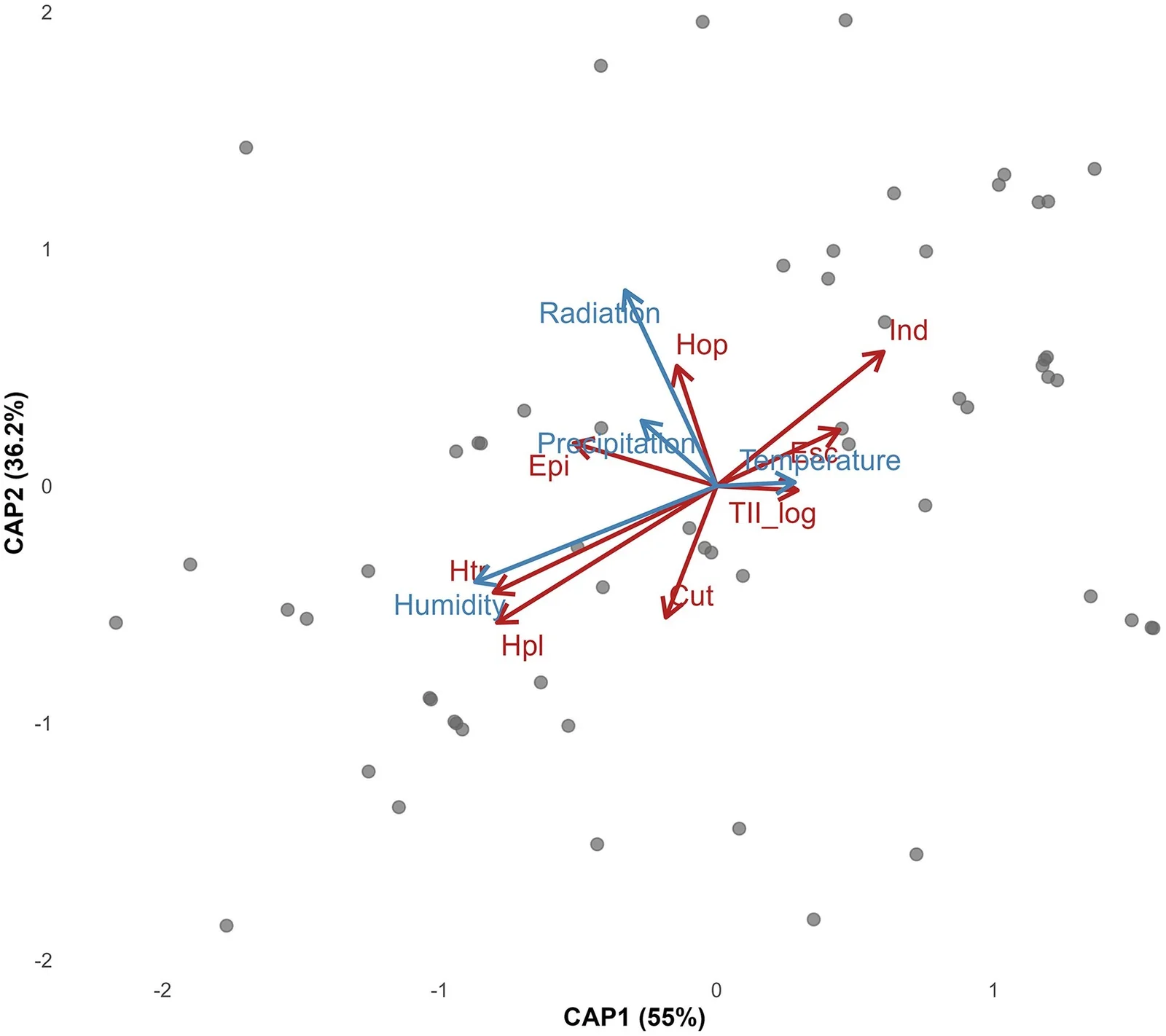
Distance-based redundancy analysis (db-RDA) showing the relationships between seven gall anatomical traits plus the log-transformed tissue investment Index (red arrows: Indu = indumentum; Cut = cuticle thickness; Epi = epidermis thickness; Esc = sclerenchyma; Htr = hypertrophy; Hpl = hyperplasia; Hop = parenchymal homogenization; TII = tissue investment) and four standardized environmental predictors (blue arrows: precipitation, temperature, radiation, humidity). The horizontal axis (CAP1; 55.0% of explained constrained variance) represents a dry–moisture gradient, with negative scores (left) associated with cellular proliferation (hypertrophy, hyperplasia) in drier sites and positive scores (right) associated with external defences (indumentum, sclerenchyma, TII) in more humid sites. The vertical axis (CAP2; 36.2%) reflects a radiation gradient, separating galls from high-irradiance environments (top)—characterized by reinforced epidermis and higher parenchymal homogenization—from those in low-irradiance environments (bottom)—characterized by thicker cuticle and higher degrees of hyperplasia and hypertrophy. Arrow length indicates the strength of the correlation; arrow direction indicates the sign (positive vs. negative) of each relationship.

In the distance-based redundancy analysis (dbRDA), we found that just over 16% of the total variation in anatomical traits could be explained by environmental variables (Table 3). Despite this modest value, the first canonical axis accounted for 55% of the explained variance (*P* = 0.003). The first canonical axis was positively associated with cellular hypertrophy and hyperplasia, and negatively associated with indumentum and sclerenchyma. The second axis explained 36.2% of the variance (*P* = 0.019) and was driven primarily by mean solar radiation, with the positive scores associated with more homogeneous parenchyma and thicker epidermis, and negative scores associated with thicker cuticle and higher degrees of hyperplasia and hypertrophy (Fig. 3). Permutation tests by the predictor confirmed that humidity (*F* = 5.05; df = 1; *P* = 0.001) and radiation (*F* = 3.85; df = 1; *P* = 0.003) were the only variables significantly associated with morphoanatomical variation.

**Table 3:** Results of the analysis of variance (ANOVA) for the redundancy analysis (dbRDA) explaining the anatomical traits and the tissue investment index (TII) based on environmental variables (precipitation, temperature, humidity and radiation).

	Df	Variance	*F*	*P*-value
(A) Global significance test of the dbRDA				
Model	4	1.819	2.734	0.001***
Residual	54	8.975		

Axis	Df	Variance	*F*	*P*-value
(B) Axis-wise significance test of the dbRDA				
CAP1	1	1.000	6.018	0.003**
CAP2	1	0.659	3.961	0.019*
CAP3	1	0.123	0.741	0.890
CAP4	1	0.036	0.215	0.983
Residual	54	8.975		

Term	Df	Variance	*F*	*P*-value
(C) Marginal effects of terms				
Humidity	1	0.839	5.053	0.001***
Radiation	1	0.640	3.850	0.003**
Precipitation	1	0.213	1.280	0.234
Temperature	1	0.137	0.822	0.551
Residual	54	8.975		

In the comparison of candidate Gamma models with a log link function, models including random effects for gall–inducer family, host–plant family, or habitat showed AIC values very similar to those of the generalized linear model (ΔAIC < 2; see Supplementary Table S1), whereas the full mixed-effects model showed substantially weaker support. Therefore, the simpler generalized linear model containing only humidity and solar radiation as fixed effects was retained for subsequent analyses. Because our dataset combines multiple plant–gall systems and direct partitioning of within-versus between-species variance was not feasible (co-occurring species across the sampled environmental gradient were not available), we used the random-effect estimates as an indirect test of taxonomic confounding. Gall–inducer family and host–plant family explained negligible variance (0.001 and <0.001, respectively), indicating that the humidity, TII relationship is not primarily driven by family-level taxonomic identity. We adopted family as the finest taxonomic level available for this comparative dataset because phylogenetic resolution for the gall–inducer taxa remains insufficient for a reliable species-level random-effects structure. Residual diagnostics indicated a good overall model fit, with no evidence of systematic patterns between residuals and fitted values. No relevant collinearity was detected among environmental predictors, indicating statistical independence among their estimated effects.

**Figure 4:**
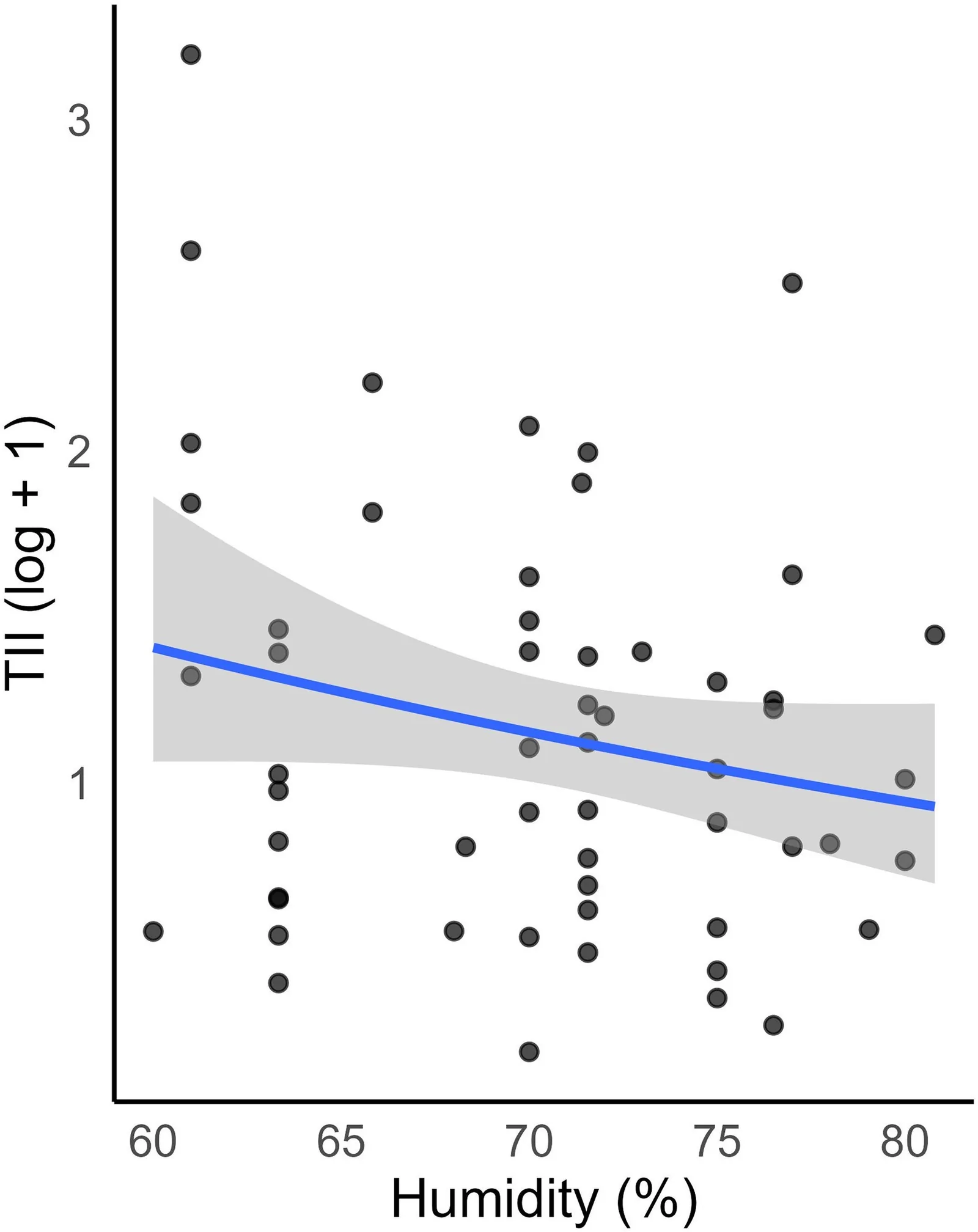
Relationship between relative humidity and the gall tissue investment index (TII, logarithm-transformed). The points represent individual samples; the solid line is the adjusted gamma-log regression (GLM) with a 95% confidence interval. A clear negative trend indicates that higher humidity is associated with lower investment in the outer gall compartment.

Our results showed a consistent negative association of relative humidity with TII and represented the main environmental predictor associated with variation in anatomical investment patterns. Higher humidity values were associated with lower TII values (coefficient = −0.024; SE = 0.012; *P* = 0.05; 95% CI: −0.046 to −0.001). Based on the fitted model, a one-percentage-point increase in relative humidity corresponded to an approximate 2.3% reduction in TII (Fig. 4).

## Discussion

Our study provides an integrative assessment of how multiple gall anatomical traits respond to environmental gradients, showing that air humidity and solar radiation emerged as the environmental variables most consistently associated with morphoanatomical variation in our dataset. However, a substantial proportion of trait variation remained unexplained by the climatic predictors included in our analyses, suggesting that additional factors such as host–plant identity, inducer biology, and local ecological conditions continue to be understood as the main drivers of diversity, as suggested by Ferreira et al. (2019). These gradients influence the relative investment in internal and external tissue compartments of galls, reflecting coordinated shifts in nutrition- and protection-related traits. Such patterns are consistent with environmentally associated anatomical variation as described for the other plant function responses to environmental stress (Franks and Farquhar 2007, Nicotra et al. 2010, Gratani 2014) and suggest that galls are functional plant organs capable of responding to environmental cues (*sensu* Palacio-López et al. 2015), even though our comparative, multi-species dataset does not allow this variation to be attributed to within-genotype phenotypic plasticity in the strict sense.

Revisiting the defence–nutrition trade-off hypothesis, our results are broadly consistent with Fernandes et al. (2026) and suggest two contrasting morphoanatomical profiles distributed along humidity and radiation gradients. The first canonical axis of the dbRDA reflects a gradient in air humidity, the variable retained as a significant predictor in our models, distinct from total precipitation, with galls from more humid environments exhibiting higher degrees of cellular hypertrophy and hyperplasia, while those from drier environments showed greater development of indumentum and sclerenchyma. The second canonical axis reflects a radiation gradient, distinguishing galls from high-irradiance environments, characterized by more homogeneous parenchyma and thicker cuticle, from those under lower radiation, which showed lower degrees of hyperplasia and hypertrophy. While traits such as the degree of hyperplasia and hypertrophy of storage tissues, the presence of indumentum and sclerenchyma, and variation in the TII respond to humidity levels, cuticle and epidermis thickness and parenchyma homogenization show stronger associations with radiation gradients.

These findings suggest that environmental factors are associated with recurrent anatomical patterns across plant–gall systems, highlighting a broader ecological dimension of gall morphology. Rather than being exclusively interpreted as an inducer-controlled extended phenotype (Stone and Schönrogge 2003), gall structure emerges from complex interactions among plant development, inducer activity, and environmental conditions. Within this broader framework, our analyses specifically explored whether broad environmental gradients were associated with variation in gall anatomical traits across diverse plant–gall systems. Our results thus contribute to the debate on the adaptive significance of gall morphology and distribution (Fernandes and Price 1991, Stone and Schönrogge 2003) by examining anatomical patterns from a comparative perspective.

Our results suggest that functional anatomical traits and their trade-offs may contribute to understanding how gall systems vary across environmental contexts. Because gall anatomy likely reflects interactions among environmental conditions, host–plant characteristics, and inducer biology, the observed patterns should be interpreted as one component of a broader multidimensional framework shaping gall structure. Recurrent associations between similar environmental conditions and anatomical traits may indicate convergent patterns across plant–gall systems, consistent with the polyphyletic and repeatedly evolved nature of the galling habit across multiple lineages (Harris and Pitzschke 2020).

Given the literature-based and correlational nature of our dataset, this study is best understood as a hypothesis-driven synthetic perspective supported by exploratory comparative analyses. Therefore, the observed relationships should be interpreted as associations that generate mechanistic hypotheses for future studies rather than as direct evidence of environmental determination of gall anatomy.

### Environmental gradients and gall anatomical variation across plant–gall systems

Several host plants in our dataset supported multiple gall morphotypes, underscoring the importance of multi- and superhost plants as drivers of anatomical diversity (Arriola and Isaias 2021, Aguilera et al. 2022). The taxonomic breadth of the systems analysed reinforces that the patterns detected here are not restricted to a single host lineage. Environmental conditions further shape gall traits through ecological processes such as microhabitat variation, soil conditions, inducer biology, and natural enemy pressure (Luz et al. 2021, Arriola et al. 2024), processes that are ultimately reflected in gall anatomy, as shown by the present analyses. It is worth mentioning that the current dataset is predominantly composed of Cerrado and Atlantic Forest systems, and the relationships observed should be interpreted primarily within the context of these environments. Regions such as the Amazon Basin, Pantanal, and Pampas remain comparatively under-sampled (Julião et al. 2017, Araújo et al. 2019, Proença and Maia 2023); expanding standardized anatomical analyses across these regions will be essential to test whether the patterns identified here hold at broader Neotropical scales.

### Environmental gradients and gall strategies to withstand stress

The dermal system represents the first interface between galls and the external environment, playing important roles in mechanical defence, water balance and gas exchange (Isaias et al. 2025), consistent with the view of galls as extended phenotypes shaped to protect the inducer against external abiotic stress (Stone and Schönrogge 2003). In our study, galls from low-humidity environments frequently exhibited dense indumentum, thickened cuticle or epidermis. Trichomes may contribute to mechanical protection and to reduced evapotranspiration, while also accumulating phenolic compounds that act as antioxidants and UV filters (Moura et al. 2008, Kar et al. 2013, Huang et al. 2015, Ferreira et al. 2019, Nóbrega et al. 2023, Guedes et al. 2024). At the same time, dense trichomes and thickened cuticle may reduce O_2_ and CO_2_ diffusion (Castro et al. 2012, Huang et al. 2015, Pincebourde and Casas 2016), indicating that these dermal traits balance desiccation protection with metabolic requirements. Galls can compensate for such structural constraints through physiological adjustments, such as localized photosynthesis or modifications in stomatal structure and function (Carneiro et al. 2017, Castro et al. 2023), highlighting that dermal traits are interpreted alongside ground tissues and internal metabolic regulation for understanding gall functioning.

Tissue hyperplasia and cell hypertrophy, two of the most characteristic anatomical features of galls, were associated in our analyses with more humid environments. These processes promote the redifferentiation of ground parenchyma into common storage tissues (CST), which function as reservoirs of water and metabolites supporting gall growth and inducer nutrition (Ferreira et al. 2019, Costa et al. 2022, Jorge et al. 2022). Increased cell division followed by expansion results in compact tissues capable of retaining water and nutrients while stabilizing the gall microenvironment (Bragança et al. 2017, Guimarães et al. 2021). Such tissues also accumulate primary and secondary metabolites that contribute to metabolic regulation and defence within the gall (Guedes et al. 2025, Kuster et al. 2025).

In contrast, galls from drier environments were characterized by sclerenchymatous tissues with thick-walled lignified cells that surround the internal compartment, providing structural support and acting as barriers that reduce water loss while increasing mechanical resistance against cecidophages and parasitoids (De Bruyn et al. 1998, Mendonça and Romanowski 2002, Stone and Schönrogge 2003, Bragança et al. 2020, Costa et al. 2021, 2022, Nogueira et al. 2022). The differentiation of sclerenchymatous cells is also associated with oxidative stress generated both by inducer activity and by abiotic water deficit, with reactive oxygen species being consumed and immobilized within the cell wall during lignin formation (Isaias et al. 2015, Fernandes et al. 2026). Although energetically costly, this process contributes to the stabilization of the gall microenvironment and supports larval development under stressful conditions (Ferreira et al. 2019).

Water and nutrients reach galls through vascular tissues, which also exhibit considerable plasticity during gall development and support the metabolic demands of the inducer. Previous studies have shown that vascular reorganization may enhance the supply of water, minerals, and photoassimilates to gall tissues, reinforcing the role of galls as strong physiological sinks within the host plant (Oliveira et al. 2016, Jorge et al. 2022, Bragança et al. 2025). Although we did not quantify vascular investment directly, such modifications may help sustain gall growth and internal homeostasis under fluctuating environmental conditions, potentially modulating the balance between defensive and nutritive roles of outer and inner tissue compartments (Aguilera et al. 2022).

**Figure 5:**
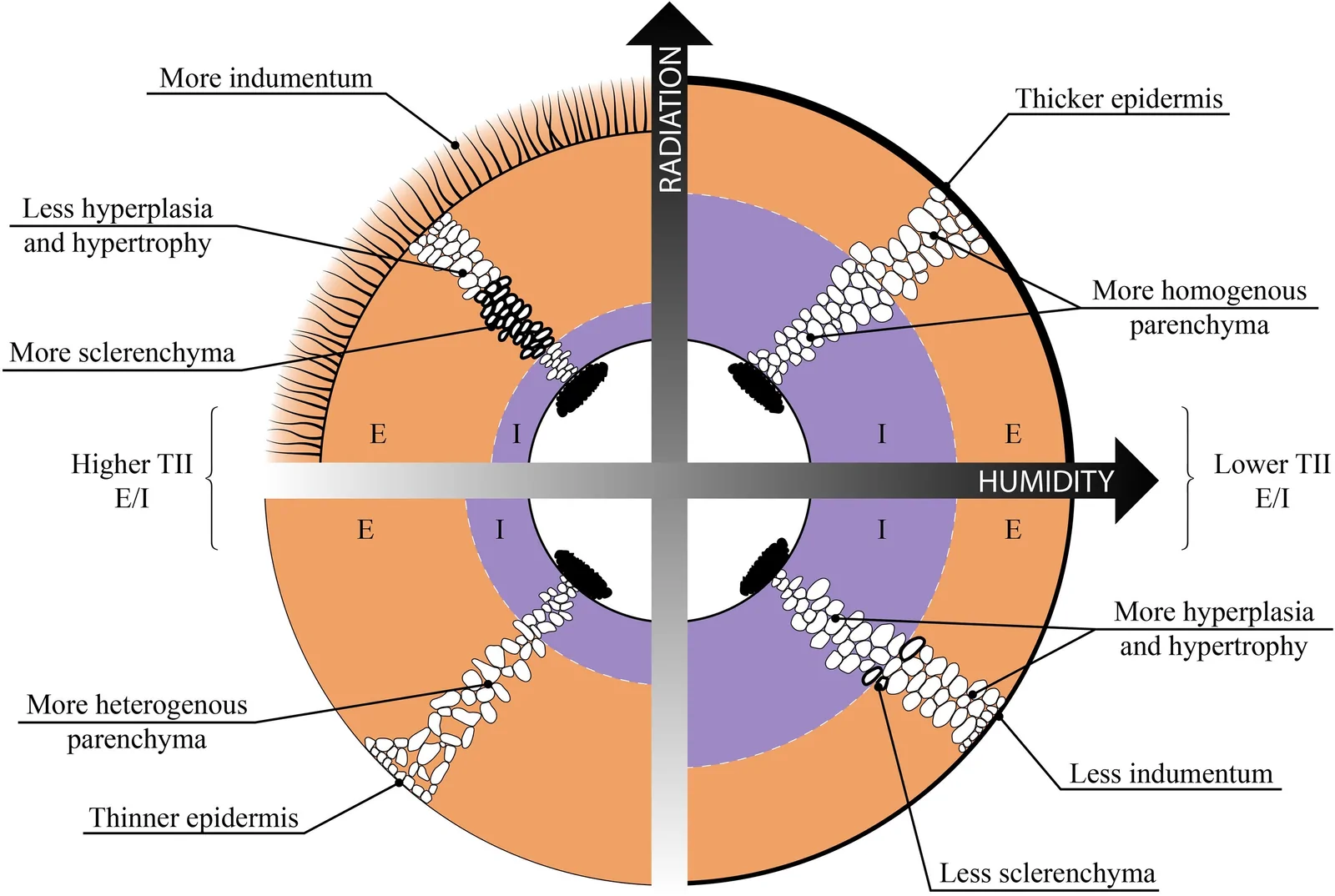
Conceptual model of gall anatomical trait syndromes distributed along orthogonal gradients of radiation (vertical axis) and humidity (horizontal axis), derived from multivariate ordination analyses. Each quadrant represents a distinct combination of environmental conditions and associated anatomical traits, including variation in epidermis/cuticle thickness, trichome density, sclerenchyma development, degree of hyperplasia and hypertrophy, and parenchyma organization. The different tissue investment is shown by the thickness of the compartments along the humidity axis. Each quadrant represents the scenarios resulting from our analyses. E = external tissues; I = internal tissues; TII = tissue investment index.

Based on the ordination results, our analyses suggest that distinct combinations of gall anatomical traits are associated with contrasting environmental gradients. Along the ordination space, combinations of high radiation and low humidity were associated with galls exhibiting dense indumentum, sclerenchymatous reinforcement, and reduced hyperplasia and hypertrophy, consistent with increased mechanical protection and mechanisms of stress dissipation and water conservation (Fig. 5, upper left quadrant). In contrast, combinations of low radiation and high humidity were associated with galls that tend to exhibit less indumentum and sclerenchyma, together with higher degrees of hyperplasia and hypertrophy, resulting in a well-developed cortex fomented by greater water availability (Fig. 5, bottom right quadrant). Intermediate combinations of these gradients are associated with thicker epidermis and a higher degree of parenchyma homogenization, balancing water retention with metabolic capacity under high radiation and humidity (Fig. 5, upper right quadrant), while under low radiation and humidity, galls are expected to display thinner epidermis and heterogenous parenchyma, most similar to non-galled organs (Fig. 5, bottom left quadrant). The univariate analysis reinforces the role of the hydric gradient, indicating that an increasing humidity gradient is associated with decreasing values of TII. This pattern reflects a shift in the balance between protective and nutritive traits, with humid conditions favouring internal tissue expansion (through hyperplasia, hypertrophy, and parenchyma homogenization) over the reinforcement of external tissues (via sclerenchyma and trichome overdifferentiation). This finding corroborates the internal–external tissue investment trade-off by Fernandes et al. (2026), and extends it by incorporating a broader range of plant–gall systems across diverse habitats. The negative association between humidity and TII suggests that galls in more humid environments tend to allocate relatively less structural investment to external tissue compartments, reflecting a shift in the defence–nutrition trade-off towards the internal compartment.

Although TII varied significantly along environmental gradients, it did not differ significantly among the morphoanatomical groups identified by hierarchical clustering. This result suggests that tissue investment patterns may be better represented as continuous responses to environmental variation than as sharply discrete categories. The identified groups were structured by the joint variation of multiple anatomical traits, particularly hyperplasia and hypertrophy, whereas TII represented only one component of the broader anatomical variation.

These patterns suggest that gall anatomy may reflect recurring variation in morphofunctional traits to environmental gradients, structured primarily by water availability and modulated by solar radiation. Radiation is associated with trait variations such as epidermal thickness and tissue organization, while previous studies indicate additional effects on gall abundance, size, and metabolic features, including pigment accumulation and photosynthetic activity (Bomfim et al. 2019, Castro et al. 2023). Because light exposure varies substantially across natural habitats and even within individual plants, further studies are needed to better understand how light quality and intensity shape gall anatomical traits on broader scales (e.g. community level).

### Anatomical variation across gall systems and its potential as an environmental indicator

Phenotypic plasticity in plants is widely recognized as a key adaptive strategy that enables structural and functional responses to environmental variation, thereby enhancing resilience and reproductive success (Sultan 2000, Palacio-López et al. 2015). A similar pattern appears to occur in galls, although their development results from the interaction between host plant tissues and gall-inducing organisms. Consequently, gall phenotypes may reflect complex physiological adjustments involving both plant responses and inducer manipulation in response to environmental stressors (Ferreira et al. 2019, Fernandes et al. 2026).

These responses illustrate how environmentally associated anatomical variation mediates the dual challenges imposed by gall-inducing organisms and environmental stressors. In plants, a thicker cuticle or epidermis is typically associated with drought resistance (Givnish 2002, Gratani 2014). However, our results indicate that these traits are not restricted to the most stressful environments, suggesting that gall structures adjust their anatomy along environmental gradients through a defence–nutrition trade-off. In this context, this anatomical adjustment allows galls to modulate both the structural investment (Fernandes et al. 2026) and the physiological changes (Ferreira et al. 2019, Arriola et al. 2024) of their tissues, optimizing inducer survival under contrasting climatic regimes. The interaction between plant developmental responses, inducer activity, and environmental conditions likely explains the substantial morphological variation observed among gall systems (Isaias et al. 2012), reinforcing the view that gall structure reflects dynamic responses to both biotic and abiotic pressures within specific ecological niches.

Because gall morphology also reflects plant responses to environmental conditions, these structures may provide valuable insights into ecosystem quality. Changes in gall diversity and morphology have been associated with environmental disturbances and habitat alteration (Santana and Isaias 2014, Toma et al. 2014, Brito et al. 2018, Costa and de Araújo 2019). Consequently, gall traits can function as sensitive bioindicators of environmental change, particularly when their anatomical composition and structural complexity are considered. In this sense, analysing gall anatomy provides a functional-trait framework for understanding how gall morphology mediates ecological interactions and ecosystem processes. By quantifying anatomical traits, it becomes possible to relate gall form and function in a manner analogous to trait-based approaches used in plant ecology (Westoby and Wright 2006, Pérez-Harguindeguy et al. 2013). Such perspectives help clarify the mechanistic basis of gall plasticity and reinforce the value of gall traits as indicators of environmental integrity.

Our dataset is derived from published histological images produced under varying technical conditions. Although we applied standardized scoring criteria (Table 2) and minimized observer bias through independent double-coding, residual methodological heterogeneity among source studies cannot be entirely excluded as a source of variation in the anatomical traits analyzed. Likewise, climatic information was compiled from different environmental databases with distinct temporal structures, combining collection-year meteorological observations for Brazilian localities and long-term climatic averages for international sites. Although all environmental variables were standardized prior to analyses and represented equivalent climatic descriptors, differences in temporal resolution may introduce additional uncertainty that cannot be fully controlled in literature-based comparative analyses.

This study expands our understanding of gall developmental responses to environmental gradients by highlighting the role of these gradients in shaping gall anatomy and related ecological strategies. Galls from dry, high-radiation environments tend to share traits associated with structural reinforcement and water conservation, whereas galls from humid, low-radiation environments exhibit anatomical profiles that favour storage tissues and metabolic regulation. These recurrent patterns across plant–gall systems suggest gall anatomical traits may have potential as indicators of environmental conditions. Future studies integrating standardized anatomical traits and morphofunctional tissue compartments across broader spatial and temporal scales should provide novel insights into the ecologically complex nature of gall structure and its responsiveness to environmental variability.

## Supplementary Material

plag038_Supplementary_Data

## Data Availability

Raw data and R code are available online at https://doi.org/10.5281/zenodo.22017281
