# Supplementary material for: Revisiting gall anatomy through a functional-trait approach across environmental gradients
Source: AoB Plants. 2026 Aug 27;18(5):plag038. doi: 10.1093/aobpla/plag038 (PMC13564390; doi:10.1093/aobpla/plag038)
Supplement: Supplementary file 1 [file plag038_supplementary_data.docx]

## **SUPPLEMENTARY MATERIAL**

Table S1. Model selection results comparing candidate models used to evaluate environmental predictors of Tissue Investment Index (TII). Models including single random effects showed similar support to the fixed-effects model (ΔAIC < 2), whereas the full mixed model showed substantially weaker support. Variance estimates associated with some random effects were negligible; therefore, the simpler GLM was retained for subsequent analyses.

| **Model** | **Fixed effects** | **Random effects** | **AIC** | **ΔAIC** |
| --- | --- | --- | --- | --- |
| GLM | Humidity + Radiation | - | 99.34 | 0.00 |
| GLMM | Humidity + Radiation | Gall-inducer family | 101.27 | 1.93 |
| GLMM | Humidity + Radiation | Host-plant family | 101.28 | 1.94 |
| GLMM | Humidity + Radiation | Habitat | 101.28 | 1.94 |
| GLMM | Humidity + Radiation | Gall-inducer family + Host-plant family + Habitat | 105.27 | 5.93 |
